# Spontaneous echo contrast in the left atrial appendage is linked to a higher risk of thromboembolic events and mortality in patients with atrial fibrillation

**DOI:** 10.1016/j.ijcha.2024.101590

**Published:** 2024-12-28

**Authors:** Jan Traub, David Hettesheimer, Jule Pinter, Floran Sahiti, Georg Fette, Carsten Henneges, Caroline Morbach, Sebastian Herrmann, Frank Puppe, Anna Frey, Stefan Störk, Martin Christa

**Affiliations:** a Department of Internal Medicine I, University Hospital Würzburg, Würzburg, Germany; b Department of Clinical Research and Epidemiology, Comprehensive Heart Failure Center, University and Hospital Würzburg, Würzburg, Germany; c Service Center Medical Informatics (SMI), University Hospital Wurzburg, Würzburg, Germany; d Caritas Hospital Bad Mergentheim, Bad Mergentheim, Germany

**Keywords:** Atrial fibrillation, Transesophageal echocardiography, Thrombus, Spontaneous echo contract, Mortality

## Abstract

**Background:**

Cardioversion, a rhythm control treatment for atrial fibrillation (AF), requires ruling out cardiac embolic sources, often originating from the left atrial appendage (LAA). Transesophageal echocardiography (TEE) is used for LAA evaluation, but it is invasive and not widely available. This study aimed to identify cardiovascular risk factors linked to LAA abnormalities and predictors of thromboembolic events and all-cause mortality.

**Methods:**

A single-center retrospective analysis included AF patients admitted to the University Hospital Würzburg between 2009 and 2018 undergoing TEE.

**Results:**

Among 2400 AF patients (median age 72; 36 % women), 469 (20 %) had LAA abnormalities: 282 (60 %) had spontaneous echo contrast (SEC), 72 (15 %) had thrombus formation (THR), and 115 (25 %) had both. Predictors of LAA abnormalities included age (OR 1.04; p = 0.002), congestive heart failure (OR 1.70; p = 0.009), diabetes (OR 1.74; p = 0.007), stroke history (OR 3.36; p = 0.001), vascular disease (OR 1.57; p = 0.026), elevated alkaline phosphatase (OR 1.15; p = 0.003), prior VKA intake (OR 1.53; p = 0.002), and DOAC intake (OR 0.57; p = 0.038). SEC with or without THR independently predicted thromboembolic events (HR 1.74, p = 0.031 and HR 1.53, p = 0.006) and all-cause mortality (HR 1.77, p = 0.011 and HR 1.57, p = 0.002), adjusted for cardiovascular risk factors, anticoagulation, and laboratory data.

**Conclusions:**

In AF patients undergoing TEE, SEC, often overlooked in cardioversion decisions, independently predicted thromboembolic events and mortality.

## 1 Introduction

Atrial fibrillation (AF) is the most prevalent cardiac arrhythmia, affecting 2–4 % of adults in developed countries and millions globally [1]. The incidence of AF is rising as a result of demographic shifts, including an aging population and the growing importance of cardiovascular risk factors. By 2030, the European Union is projected to have 14–17 million individuals with AF, with 120,000–215,000 new cases annually [2]. This trend underscores the growing burden of AF on healthcare systems and highlights the need for effective patient management and strategies to identify patients at risk for additional adverse events.

Cardioversion represents a key aspect of rhythm control for patients with AF, with the objective to restore a regular sinus rhythm. Prior to cardioversion, current practice guidelines recommend transesophageal echocardiography (TEE) [3] to detect left atrial appendage (LAA) abnormalities such as spontaneous echo contrast (SEC) or thrombus formation (THR). This is of particular relevance in patients with AF lasting more than 48 h or of an unknown duration, and/or who have been taking their medication inconsistently or have previously received inadequate anticoagulation therapy. The objective of a TEE evaluation is to either minimize the risk of a stroke or to prevent a stroke from occurring, both during and after the cardioversion procedure. The evidence of THR has significant impact on decisions regarding anticoagulation therapy and timing of cardioversion [4]. These abnormalities are linked to blood stasis and hypercoagulability in the LAA, underscoring the importance of their identification for effective management and risk reduction in AF patients. Of note, the presence of SEC alone has no therapeutic consequences. While some studies have discussed the predictive significance of the CHA_2_DS_2_-VASc score in the occurrence of abnormalities in the LAA [5], [6], the associations of cardiovascular risk factors with the presence LAA abnormalities in patients with AF are incompletely understood. Further, the relevance of LAA abnormalities as an independent predictor of thromboembolic events and mortality risk is unknown.

We therefore aimed to determine in a large sample of patients hospitalized with AF the association of cardiovascular risk factors and routine laboratory parameters with LAA abnormalities. Second, we estimated the prognostic utility of SEC or THR as independent predictors of thromboembolic events and increased mortality risk. We hypothesized that in these patients the presence of LAA abnormalities is a) determined by cardiovascular risk factors and comorbidities, and b) independently predicts thromboembolic events and an increased risk of all-cause mortality.

## 2 Methods

### 2.1 Ethical considerations and data warehouse

We retrospectively analyzed data derived from the Clinical Data Warehouse of the University Hospital Würzburg. The Data Warehouse is a core facility of the Hospital that facilitates storage of all routinely collected electronic patient information through various medical record systems and enables data linkage of individual subjects over time [7], [8]. The Data Warehouse is operational through procedures supervised by the Hospitaĺs data protection officer, and approved by the Ethics Committee of the University Hospital Würzburg (# 02022015).

### 2.2 Patient selection and data extraction

Within the study period between January 1, 2009, and November 30, 2018, adult patients with incident or prevalent AF were eligible if they had undergone TEE during their hospital stay. Age, sex, coded medical diagnoses, and the first available laboratory results were identified and exported. Additionally, available survival data (i.e. time from index hospital admission until death or last known visit) and information on future thromboembolic events (I63 and I74) was derived and exported. Patients with incomplete data were excluded (n = 84), resulting in a final dataset of 2400 patients for analysis.

The following components of the CHA_2_DS_2_-VASc-score were identified using ICD-10 diagnosis codes available in the patient records: Congestive heart failure (I50.x), hypertension (I10.x-I15.x), diabetes mellitus (E10.x-E14.x), history of stroke or transient ischemic attack (TIA; I63.x, I64.x, and G45.x), and vascular disease (I70.x, I71.x, and I73.x).

### 2.3 Transesophageal echocardiography

According to local standards, patients with insufficient or unclear anticoagulation status routinely undergo TEE prior to pharmacological or electrical cardioversion. This group of patients was identified using the Data Warehouse approach and entered the present analyses, as well as any other patient undergoing TEE due to other medical reasons and exhibiting AF. TEE was performed within clinical routine. The standardized assessment included the dedicated evaluation of LAA structure and function (operationalized via pulsed wave Doppler emptying velocity) and the search for the presence of SEC or THR, respectively. Any TEE information addressing certain or uncertain LAA abnormalities was re-evaluated by a reader with more than 10 years of experience in echocardiography. If the uncertainty remained, another experienced reader was consulted.

### 2.4 Data analysis

Data are presented as count (percent) or median (quartiles). Analysis of variance with Bonferroni or Dunnett-T3 post-hoc tests was used to compare continuous variables between groups, and chi-squared and Fisher's exact tests were used for categorical data. Uni- and multivariable binomial logistic regression models were employed to evaluate the correlates of LAA findings, and odds ratios (OR) with 95 % confidence intervals (CI) are reported. To identify predictors of thromboembolic events and mortality risk, uni- and multivariable Cox proportional hazards regression analyses were performed, and hazard ratios (HR) with 95 % CI are reported. The proportionality assumption was checked visually and was not violated. Continuous variables (except age) were converted to z-scores to standardize their scales, allowing for easier comparison of effect sizes and ensuring that differences in measurement units did not influence model coefficients. All statistical tests were conducted two-tailed, and the statistical software SPSS (version 26) and R (version 4.4.0) was used.

## 3 Results

### 3.1 Patient characteristics

The baseline characteristics of the total sample of 2400 AF patients, categorized by TEE-derived LAA findings, are described in Table 1. In the majority of patients (80 %), no LAA abnormality was detected, while 20 % of patients exhibited LAA abnormalities (SEC or THR). Patients with any LAA abnormality were significantly older compared to those with no findings, while there was no significant difference in the distribution of sex among groups. More than half of the cohort had previously known AF and patients with LAA abnormalities were more likely to have a prior history of AF. Congestive heart failure, hypertension, diabetes mellitus, history of stroke or transient ischemic attack, and vascular disease were more prevalent in patients with LAA abnormality compared to those with no finding. Likewise, values for the CHA_2_DS_2_-VASc score were higher in patients with LAA abnormality, compared to those without. 38 % of the cohort had previously received oral anticoagulation. Interestingly, LAA findings associated with a higher frequency of vitamin K antagonist (VKA) intake, but also with a lower direct oral anticoagulant (DOAC) intake.

**Table 1:** **Descriptive statistics according to echocardiographic left atrial appendage finding.** LAA = left atrial appendage, SEC = spontaneous echo contrast, THR = thrombus formation, TIA = transient ischemic attack. Bold p values indicate significance (p value < 0.05) ^a^ no significant differences in post-hoc tests.

	**Total sample**	**No LAA abnormality**	**Any LAA abnormality**	**p value**	**SEC and THR**	**SEC,** **no THR**	**THR,** **no SEC**	**p value**
Number of patients	2400 (100)	1931 (80)	469 (20)		115 (5)	282 (12)	72 (3)	
Age (years)	72 (63–79)	71 (62–78)	75 (67–81)	**<0.001**	75 (68–80)	76 (67–81)	73 (63–81)	0.698
Female sex	856 (36)	692 (36)	164 (35)	0.725	42 (37)	99 (35)	23 (32)	0.813
Previously known atrial fibrillation	1286 (54)	998 (52)	288 (61)	**<0.001**	75 (65)	180 (64)	33 (46)	**<0.001**
Congestive heart failure (I50.x)	1036 (43)	792 (41)	244 (52)	**<0.001**	57 (50)	142 (50)	45 (63)	0.153
Hypertension (I10.x-I15.x)	1786 (74)	1417 (73)	369 (79)	**0.018**	92 (80)	277 (81)	50 (69)	0.114
Diabetes mellitus (E10.x-E14.x)	584 (24)	437 (23)	147 (31)	**<0.001**	35 (30)	92 (33)	20 (28)	0.710
History of stroke or TIA (I63.x, I64.x, G45.x)	190 (8)	142 (7)	48 (70)	**0.038**	10 (9)	33 (12)	5 (7)	0.405
Vascular disease (I70.x, I71.x, I73.x)	801 (33)	612 (32)	189 (40)	**<0.001**	52 (45)	101 (36)	36 (50)	**0.042**
CHA_2_DS_2_-VASc Score	3 (2–5)	3 (2–5)	4 (3–5)	**<0.001**	4 (3–5)	4 (3–5)	4 (3–5)	0.537
Erythrocytes (10^3^/µl)	4.6 (4.1–4.9)	4.6 (4.1–4.9)	4.5 (4.1–4.9)	0.111	4.5 (4.2–4.9)	4.5 (4.0–4.9)	4.5 (4.0–5.0)	0.941
Hemoglobin (g/dl)	14 (12–15)	14 (12–15)	14 (12–15)	0.273	13 (12–15)	14 (12–15)	14 (12–15)	0.556
Hematocrit (%)	41 (37–44)	41 (37–44)	40 (37–44)	0.810	40 (37–43)	41 (36–44)	41 (37–44)	0.542
Mean cellular volume (fl)	89 (86–93)	89 (86–92)	90 (87–93)	**<0.001**	89 (86–92)	90 (87–93)	91 (87–95)	0.080
Mean cellular hemoglobin (pg)	30 (29–31)	30 (29–31)	30 (29–32)	0.364	30 (28–31)	30 (29–32)	30 (29–32)	0.111
Mean corpuscular hemoglobin concentration (g/dl)	34 (33–35)	34 (33–35)	34 (33–34)	**0.001**	33 (33–34)	34 (33–34)	34 (33–34)	0.886
Leucocytes (10^3^/µl)	7.4 (6.1–9.4)	7.4 (6.0–9.3)	7.6 (6.4–9.6)	0.716	8.0 (6.9–10.2)	7.6 (6.3–9.3)	7.2 (6.1–9.0)	0.147
Thrombocytes (10^3^/µl)	215 (177–261)	216 (179–264)	205 (170–253)	**0.003**	210 (184–259)	203 (161–253)	198 (166–244)	0.177
Estimated glomerular filtration rate (ml/min/1.73 m^2^)	67 (50–82)	68 (51–84)	61 (43–77)	**<0.001**	63 (48–80)	61 (41–77)	58 (45–75)	0.421
Alanine transaminase (U/l)	24 (18–37)	24 (18–36)	25 (17–39)	0.462	24 (18–37)	25 (17–38)	25 (17–48)	**0.002^a^**
Aspartate transaminase (U/l)	28 (22–36)	27 (22–35)	30 (24–39)	0.297	31 (25–40)	28 (24–38)	33 (23–47)	**0.003^a^**
Alkaline phosphatase (U/l)	74 (60–92)	74 (59–89)	78 (62–105)	**<0.001**	82 (64–102)	76 (60–102)	86 (67–122)	0.745
Urea (mg/dl)	39 (31–54)	38 (30–52)	44 (34–62)	**<0.001**	44 (33–62)	43 (32–63)	42 (36–62)	0.927
Previous oral anticoagulation	910 (38)	690 (36)	220 (47)	**<0.001**	53 (46)	140 (50)	27 (38)	0.179
Vitamin K antagonists	726 (30)	525 (27)	201 (43)	**<0.001**	47 (41)	128 (45)	26 (36)	0.323
Direct oral anticoagulants	184 (8)	165 (9)	19 (4)	**<0.001**	6 (5)	12 (4)	1 (1)	0.418
Future oral anticoagulation	1656 (69)	1311 (68)	345 (74)	**0.017**	91 (79)	200 (71)	54 (75)	0.232
Vitamin K antagonists	860 (36)	620 (32)	240 (51)	**<0.001**	67 (58)	113 (47)	40 (56)	0.096
Direct oral anticoagulants	796 (33)	691 (36)	105 (22)	**<0.001**	24 (21)	67 (24)	14 (19)	0.665

Routine laboratory analysis revealed that patients with LAA abnormality had higher mean cellular volume and lower mean corpuscular hemoglobin concentration compared to those with no findings. Patients with no findings had a higher thrombocyte count compared to those without. eGFR was significantly lower and alkaline phosphatase as well as urea levels were higher in patients with LAA abnormality.

### 3.2 Independent predictors of any LAA abnormality

As shown in Table 2, significant predictors for any LAA abnormality (SEC or THR; n = 469) in the multivariable logistic regression model included age (OR per year: 1.04, 95 % CI 1.01–1.06, p = 0.002), congestive heart failure (OR 1.70, 95 % CI 1.14–2.53, p = 0.009), diabetes mellitus (OR 1.74, 95 % CI 1.17–2.61, p = 0.007), history of stroke/TIA (OR 3.36, 95 % CI 1.61–7.03, p = 0.001), vascular disease (OR 1.57, 95 % CI 1.06–2.33, p = 0.026), alkaline phosphatase levels (OR per standard deviation: 1.15, 95 % CI 1.05–1.27, p = 0.003), previous VKA intake (OR 1.53, 95 % CI 1.17–2.00; p = 0.002), and previous DOAC intake (OR 0.57, 95 % CI 0.32–0.95; p = 0.038).

**Table 2:** Predictors of any left atrial appendage abnormalities, i.e. spontaneous echocardiographic contrast and/or thrombus formation (n = 2400).

**None** (n = 1931) **vs. any LAA finding** (n = 469)	Univariable logistic regression		Multivariable logistic regression	
	OR (95 % CI)	p value	Adj. OR (95 % CI)	p value
Age (years)	**1.03 (1.02**–**1.04)**	**<0.001**	**1.04 (1.01**–**1.06)**	**0.002**
Female sex	0.96 (0.78–1.19)	0.725	1.30 (0.87–1.93)	0.198
Previously known atrial fibrillation	**1.49 (1.21**–**1.83)**	**<0.001**	1.19 (0.91–1.56)	0.200
Congestive heart failure (I50.x)	**1.56 (1.27**–**1.91)**	**<0.001**	**1.70 (1.14**–**2.53)**	**0.009**
Hypertension (I10.x-I15.x)	**1.34 (1.05**–**1.71)**	**0.019**	1.35 (0.90–2.02)	0.150
Diabetes mellitus (E10.x-E14.x)	**1.56 (1.25**–**1.95)**	**<0.001**	**1.74 (1.17**–**2.61)**	**0.007**
History of stroke or TIA (I63.x, I64.x, G45.x)	**1.44 (1.01**–**2.01)**	**0.039**	**3.36 (1.61**–**7.03)**	**0.001**
Vascular disease (I70.x, I71.x, I73.x)	**1.45 (1.18**–**1.79)**	**<0.001**	**1.57 (1.06**–**2.33)**	**0.026**
CHA_2_DS_2_-VASc-Score	**1.21 (1.14**–**1.29)**	**<0.001**	0.74 (0.54–1.02)	0.065
Erythrocytes (z score)	0.92 (0.83–1.02)	0.111	0.59 (0.08–4.26)	0.610
Hemoglobin (z score)	0.95 (0.86–1.05)	0.273	0.67 (0.03–15.25)	0.797
Hematocrit (z score)	0.99 (0.89–1.09)	0.809	2.22 (0.15–32.26)	0.557
Mean cellular volume (z score)	**1.19 (1.07**–**1.31)**	**0.001**	0.88 (0.17–5.20)	0.886
Mean cellular hemoglobin (z score)	1.05 (0.95–1.16)	0.364	1.13 (0.13–7.56)	0.908
Mean corpuscular hemoglobin concentration (z score)	**0.84 (0.76**–**0.93)**	**0.001**	0.88 (0.04–21.23)	0.938
Leucocytes (z score)	1.02 (0.92–1.12)	0.717	0.99 (0.87–1.10)	0.817
Thrombocytes (z score)	**0.85 (0.76**–**0.94)**	**0.003**	0.93 (0.82–1.04)	0.214
Estimated glomerular filtration rate (z score)	**0.79 (0.71**–**0.87)**	**<0.001**	0.99 (0.86–1.14)	0.909
Alanine transaminase (z score)	1.03 (0.93–1.12)	0.472	1.01 (0.73–1.36)	0.971
Aspartate transaminase (z score)	1.04 (0.95–1.14)	0.317	1.03 (0.75–1.38)	0.861
Alkaline phosphatase (z score)	**1.19 (1.08**–**1.33)**	**0.001**	**1.15 (1.05**–**1.27)**	**0.003**
Urea (z score)	**1.21 (1.11**–**1.33)**	**<0.001**	1.09 (0.96–1.24)	0.185
Previous vitamin K antagonists	**2.01 (1.63**–**2.47)**	**<0.001**	**1.53 (1.17**–**2.00)**	**0.002**
Previous direct oral anticoagulants	**0.45 (0.27**–**0.72)**	**0.001**	**0.57 (0.32**–**0.95)**	**0.038**

### 3.3 Subgroups of LAA abnormalities

Of all 469 patients with LAA abnormality, 115 (25 %) showed both SEC and THR, while the majority of 282 (60 %) had only SEC without THR, and 72 (15 %) exhibited only THR without SEC. As displayed in Table 1, age and sex did not differ between these subgroups. Patients with THR only had a lower frequency of previously known AF, while the frequency of patients with vascular disease was somewhat higher in patients with only THR, compared to those with only SEC. Global differences in alanine and aspartate aminotransferase levels did not retain significance after correction in post-hoc tests.

### 3.4 LAA abnormalities and thromboembolic events

**Fig. 1:**
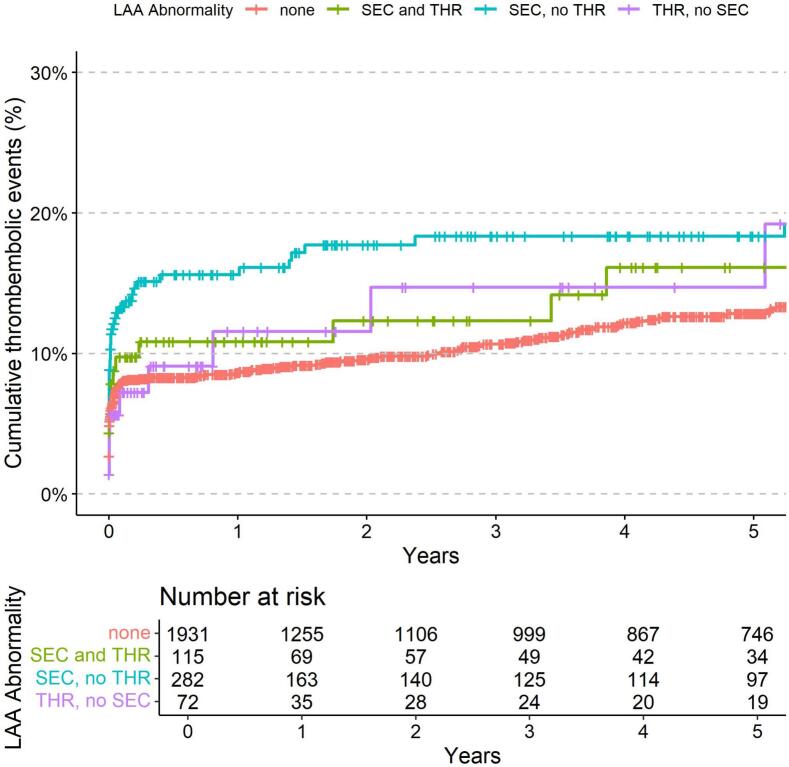
**Association of left atrial appendage (LAA) abnormalities with thromboembolic events.** The Kaplan-Meier plot illustrates the 5-year cumulative incidence for patients with atrial fibrillation undergoing transesophageal echocardiography in subgroups of LAA abnormalities. The four groups represented are: no LAA abnormality (red), spontaneous echo contrast and thrombus formation (SEC and THR; green), spontaneous echo contrast without thrombus formation (SEC, no THR; blue) and thrombus formation without spontaneous echo contrast (THR, no SEC; blue). (For interpretation of the references to colour in this figure legend, the reader is referred to the web version of this article.)

Within a median follow-up time of 3.8 years (quartiles 0.5, 7.0), 253 patients (10.5 %) had a reported thromboembolic event. While stroke (I63) was the most common thromboembolic complication (n = 233), other arterial embolism (I74) was reported in only 20 patients (8 %). As shown in Table 3, patients with SEC, but without THR had a higher risk for thromboembolic events the univariable analysis (HR 1.70, 95 % CI 1.28–2.26; p < 0.001). Fig. 1 illustrates the cumulative incidence of thromboembolic events according to LAA findings.

**Table 3:** Predictors of thromboembolic events (n = 2400).

	Univariable Cox regression		Multivariable Cox regression	
	HR (95 % CI)	p value	Adj. HR (95 % CI)	p value
Spontaneous echocardiographic contrast and thrombus	1.23 (0.77–1.99)	0.386	**1.74 (1.05**–**2.86)**	**0.031**
Spontaneous echocardiographic contrast, no thrombus	**1.70 (1.28**–**2.26)**	**<0.001**	**1.53 (1.13**–**2.07)**	**0.006**
Thrombus, no spontaneous echocardiographic contrast	1.27 (0.69–2.31)	0.441	1.40 (0.74–2.64)	0.296
Age (years)	1.01 (1.00–1.02)	0.005	1.00 (0.98–1.03)	0.848
Female sex	1.16 (0.93–1.46)	0.196	0.85 (0.55–1.29)	0.441
Previously known atrial fibrillation	0.63 (0.51–0.79)	<0.001	0.85 (0.67–1.09)	0.197
Congestive heart failure (I50.x)	0.56 (0.43–0.71)	<0.001	0.91 (0.58–1.43)	0.693
Hypertension (I10.x-I15.x)	1.65 (1.24–2.19)	0.001	0.94 (0.60–1.48)	0.781
Diabetes mellitus (E10.x-E14.x)	1.53 (1.21–1.94)	<0.001	0.81 (0.52–1.27)	0.360
History of stroke or TIA (I63.x, I64.x, G45.x)	206 (149–286)	<0.001	**172 (79**–**370)**	**<0.001**
Vascular disease (I70.x, I71.x, I73.x)	0.67 (0.52–0.87)	0.002	**0.56 (0.35**–**0.89)**	**0.014**
CHA_2_DS_2_-VASc-Score	1.56 (1.46–1.68)	<0.001	1.17 (0.83–1.64)	0.377
Erythrocytes (z score)	0.90 (0.81–1.01)	0.070	**0.12 (0.02**–**0.73)**	**0.022**
Hemoglobin (z score)	0.95 (0.85–1.07)	0.404	6.78 (0.48–95.70)	0.156
Hematocrit (z score)	0.91 (0.81–1.01)	0.082	1.30 (0.04–46.37)	0.887
Mean cellular volume (z score)	1.00 (0.90–1.12)	0.937	**0.07 (0.01**–**0.39)**	**0.003**
Mean cellular hemoglobin (z score)	1.12 (1.00–1.25)	0.047	**12.39 (1.91**–**80.49)**	**0.008**
Mean corpuscular hemoglobin concentration (z score)	1.18 (1.08–1.30)	0.001	**0.18 (0.04**–**0.74)**	**0.018**
Leucocytes (z score)	1.09 (1.04–1.15)	0.001	1.00 (0.87–1.15)	0.952
Thrombocytes (z score)	1.08 (0.97–1.20)	0.172	**1.13 (1.01**–**1.26)**	**0.039**
Estimated glomerular filtration rate (z score)	1.01 (0.90–1.12)	0.925	0.96 (0.82–1.12)	0.568
Alanine transaminase (z score)	0.40 (0.21–0.77)	0.006	0.73 (0.30–1.78)	0.487
Aspartate transaminase (z score)	0.50 (0.18–1.36)	0.174	1.01 (0.35–2.91)	0.986
Alkaline phosphatase (z score)	1.01 (0.90–1.13)	0.888	0.98 (0.87–1.10)	0.746
Urea (z score)	0.91 (0.79–1.04)	0.171	1.06 (0.88–1.28)	0.522
Vitamin K antagonist treatment	0.89 (0.70–1.12)	0.312	0.91 (0.68–1.21)	0.502
Direct oral anticoagulant treatment	0.96 (0.76–1.22)	0.748	0.91 (0.68–1.21)	0.507

When adjusting for demographics, risk factors, laboratory findings and anticoagulation status in multivariable analysis, both SEC with THR (HR 1.74, 95 % CI 1.05–2.86, p = 0.031) and SEC without THR (HR 1.53, 95 % CI 1.13–2.07, p = 0.006) remained independently predictive.

### 3.5 Prognostic utility of LAA abnormalities

**Fig. 2:**
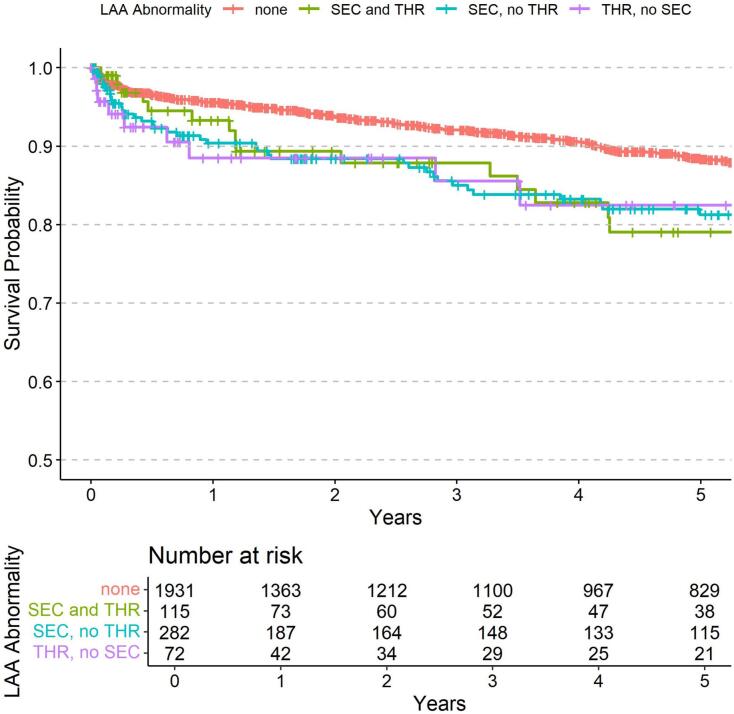
**Association of left atrial appendage (LAA) abnormalities with the probability of dying.** The Kaplan-Meier plot illustrates the 5-year survival probabilities for patients with atrial fibrillation undergoing transesophageal echocardiography in subgroups of LAA abnormalities. The four groups represented are: no LAA abnormality (red), spontaneous echo contrast and thrombus formation (SEC and THR; green), spontaneous echo contrast without thrombus formation (SEC, no THR; blue) and thrombus formation without spontaneous echo contrast (THR, no SEC; blue). (For interpretation of the references to colour in this figure legend, the reader is referred to the web version of this article.)

Within the median follow-up time of 3.8 years, 324 patients (13.5 %) had died. As shown in Table 4, the LAA subgroups SEC and THR (HR 1.72, 95 % CI 1.13–2.64, p = 0.012), as well as SEC without THR (HR 1.75, 95 % CI 1.33–2.31, p < 0.001) predicted an increased mortality risk in the univariable analysis with similar strength. To illustrate the survival probabilities over five years for patients with different LAA findings, Kaplan-Meier survival curves (Fig. 2) were calculated.

**Table 4:** Predictors of all-cause death (n = 2400).

	Univariable Cox regression		Multivariable Cox regression	
	HR (95 % CI)	p value	Adj. HR (95 % CI)	p value
Spontaneous echocardiographic contrast and thrombus	**1.72 (1.13**–**2.64)**	**0.012**	**1.77 (1.14**–**2.75)**	**0.011**
Spontaneous echocardiographic contrast, no thrombus	**1.75 (1.33**–**2.31)**	**<0.001**	**1.57 (1.18**–**2.10)**	**0.002**
Thrombus, no spontaneous echocardiographic contrast	1.48 (0.83–2.63)	0.184	1.43 (0.79–2.59)	0.236
Age (years)	**1.05 (1.04**–**1.07)**	**<0.001**	**1.04 (1.01**–**1.07)**	**0.008**
Female sex	0.87 (0.69–1.10)	0.245	0.69 (0.45–1.06)	0.091
Previously known atrial fibrillation	**1.55 (1.24**–**1.95)**	**<0.001**	1.12 (0.87–1.43)	0.382
Congestive heart failure (I50.x)	**2.75 (2.20**–**3.45)**	**<0.001**	**1.75 (1.13**–**2.71)**	**0.011**
Hypertension (I10.x-I15.x)	1.18 (0.91–1.52)	0.205	0.81 (0.53–1.24)	0.342
Diabetes mellitus (E10.x-E14.x)	**1.72 (1.36**–**2.18)**	**<0.001**	1.25 (0.82–1.90)	0.298
History of stroke or TIA (I63.x, I64.x, G45.x)	0.89 (0.57–1.40)	0.618	1.34 (0.58–3.06)	0.494
Vascular disease (I70.x, I71.x, I73.x)	**1.70 (1.37**–**2.13)**	**<0.001**	1.05 (0.69–1.61)	0.808
CHA_2_DS_2_-VASc-Score	**1.32 (1.24**–**1.40)**	**<0.001**	0.95 (0.68–1.34)	0.789
Erythrocytes (z score)	**0.58 (0.52**–**0.63)**	**<0.001**	0.66 (0.09–4.98)	0.691
Hemoglobin (z score)	**0.58 (0.53**–**0.64)**	**<0.001**	1.31 (0.06–28.37)	0.864
Hematocrit (z score)	**0.60 (0.55**–**0.67)**	**<0.001**	0.88 (0.06–13.28)	0.929
Mean cellular volume (z score)	**1.25 (1.12**–**1.41)**	**<0.001**	0.74 (0.11–4.87)	0.754
Mean cellular hemoglobin (z score)	0.95 (0.85–1.07)	0.441	1.54 (0.15–15.99)	0.716
Mean corpuscular hemoglobin concentration (z score)	**0.66 (0.59**–**0.74)**	**<0.001**	0.38 (0.02–8.64)	0.541
Leucocytes (z score)	1.09 (1.03–1.16)	0.004	1.02 (0.95–1.11)	0.524
Thrombocytes (z score)	0.93 (0.83–1.05)	0.249	1.02 (0.91–1.14)	0.753
Estimated glomerular filtration rate (z score)	**0.49 (0.43**–**0.55)**	**<0.001**	**0.69 (0.59**–**0.81)**	**<0.001**
Alanine transaminase (z score)	1.02 (0.93–1.12)	0.639	0.78 (0.58–1.04)	0.089
Aspartate transaminase (z score)	1.05 (0.99–1.12)	0.105	**1.25 (1.01**–**1.56)**	**0.044**
Alkaline phosphatase (z score)	**1.16 (1.11**–**1.20)**	**<0.001**	**1.18 (1.11**–**1.26)**	**<0.001**
Urea (z score)	**1.51 (1.42**–**1.60)**	**<0.001**	1.10 (0.98–1.23)	0.114
Vitamin K antagonist treatment	1.11 (0.89–1.39)	0.349	**0.67 (0.51**–**0.86)**	**0.002**
Direct oral anticoagulant treatment	**0.60 (0.46**–**0.79)**	**<0.001**	0.78 (0.58–1.06)	0.118

In multivariable analysis, SEC with THR (HR 1.77, 95 % CI 1.14–2.75, p = 0.011) and SEC without THR (HR 1.57, 95 % CI 1.18–2.10, p = 0.002) remained independently predictive. Other significant predictors in the multivariable model included age, congestive heart failure, estimated glomerular filtration rate, aspartate aminotransferase, alkaline phosphatase, and VKA treatment after index event.

## 4 Discussion

In this large retrospective study investigating 2400 patients with AF who also had undergone TEE, LAA abnormalities were frequent and associated with older age, higher comorbidity burden (i.e. congestive heart failure, diabetes, history of stroke or transient ischemic attack, vascular disease), and elevated alkaline phosphatase levels. As a key finding we report the independent association of isolated SEC (i.e., SEC without THR) with an increased risk of both thromboembolic events and all-cause mortality, even after adjusting for cardiovascular risk factors and anticoagulation status.

The observed frequency of LAA abnormalities (20 %) in this study is within the range of with previous studies, where SEC and/or thrombus were found in 78/641 (12 %) [9], 88/481 (18 %) [5], 81/403 (20 %) [10], 215/1001 (21 %) [11], 36/139 (26 %) [6], 116/433 (27 %) [12] and 13/36 (36 %) [13] of included AF patients. As these studies addressed different types of patient groups from different settings, these variations may be explained by the respectively different age and risk profiles. The percentage of patients with THR (8 % in our study) aligns well with another large investigation, where THR was seen in 213/2591 (8 %) of patients [14].

Numerous studies have highlighted the predictive value of the CHA_2_DS_2_-VASc score in the development of LAA abnormalities [5], [6], [12], [15], [16], [17]. However, in our analysis, the CHA_2_DS_2_-VASc score was only associated with LAA findings in the univariable analysis and did not retain this association in multivariable analysis. When we conducted a multivariable analysis, only specific components of the score—namely age, congestive heart failure, diabetes, history of stroke or transient ischemic attack, and vascular disease—were independently associated with the presence of SEC and/or thrombus in the LAA. Interestingly, sex and hypertension, which are additional components of the CHA_2_DS_2_-VASc score, did not show an independent association. This suggests that while the overall score is useful, individual risk factors within the score vary in their predictive strength for LAA abnormalities. With sex not showing an independent association in the multivariable model and therefor demonstrating a weakened predictive power, our findings support changes within the new 2024 AF guidelines of the European Society of Cardiology, where sex is removed from the CHA_2_DS_2_-VASc score, as it is considered rather a risk modifier than a risk factor [18].

Several other efforts to develop more sensitive scores to predict LAA abnormalities were published. For example, the CLOTS-AF score [11] includes the history of stroke as well as echocardiographic parameters (left ventricular ejection fraction, left atrial volume index, tricuspid annular plane systolic excursion) and creatinine levels. Others showed that patients with higher E/e’ ratios and lower e’ velocities were significantly more likely to have LAA findings, independent of traditional risk factors like the CHA_2_DS_2_-VASc score, left ventricular ejection fraction, and left atrial volume index [19], [20]. We could not confirm independent associations of renal function with LAA findings in our dataset and cannot make any statement regarding echocardiographic parameters, as these parameters were not included in our dataset.

It was interesting to see that VKA intake increased the chance for LAA abnormalities, while DOAC associated with lower risk both in uni- and multivariable analyses. These associations could be due to differences in their mechanisms, consistency of anticoagulation, and patient selection. VKAs often result in fluctuating INR levels and are typically prescribed to patients with more severe AF or higher comorbidities, inherently increasing their risk of LAA abnormalities. In contrast, DOACs provide consistent anticoagulation, potentially reducing thrombus formation and resolving existing thrombi more effectively. These findings emphasize the need for individualized anticoagulation therapy based on patient characteristics and risk factors.

The independent association of elevated alkaline phosphatase levels with LAA abnormalities is particularly intriguing. Elevated alkaline phosphatase has been linked to vascular calcification and stiffness, which might contribute to altered hemodynamics and thrombus formation in AF patients [21], [22]. More research is needed to consolidate this interesting finding and to clarify if elevated alkaline phosphatase may be incorporated into the risk assessment of AF patients.

As probably the most interesting finding of this investigation, the TEE-derived evidence of SEC and THR, as well as isolated SEC (i.e., SEC without THR) independently increased the risk for both thromboembolic events and all-cause mortality in this cohort, even after adjusting for confounding factors including anticoagulation.

Regarding thromboembolic events, it has been shown before that SEC also increases the risk of pro-thrombotic events [24], although these studies did not perform multivariable analyses. This might be an important finding for clinical practice, where the presence of SEC alone has no therapeutic consequences until now. In our study, the presence of SEC was associated with somewhat lower future anticoagulation rates, which might explain its effect on thromboembolic events. Likewise, SEC in patients with THR might suggest that anticoagulation may have been inadequate, the increased chance for thromboembolism in patients with SEC and THR may be explained by an absence of effective anticoagulation.

Regarding mortality, it is well known, e.g. from the Framingham Heart Study, that while mortality rates in AF patients with LAA thrombi are 50–90 % higher compared to those without [23], At the same time, oral anticoagulation has demonstrated efficacy in the reduction of both stroke and overall mortality [25] − known to be particularly associated with thromboembolic events [26]. The increased mortality in patients with SEC only could thus be attributed to the lack of appropriate anticoagulation. However, in our cohort, patients with SEC had only slightly lower anticoagulation rated compared to those with THR. At this point, it must be noted that our clinical practice mandates to ensure initiating (oral) anticoagulation in patients with AF as part of routine care according to current guidelines anyway [18]. Our findings suggest that monitoring and adjusting anticoagulation therapy in patients with SEC alone or SEC and THR might be crucial for improving outcomes. Early detection and intervention could potentially reduce the risk of thromboembolic events and improve overall survival rates in these patients.

Importantly, SEC might also reflect significantly impaired atrial function. SEC, which neither is considered a contraindication to electrical cardioversion nor influences anticoagulation decisions, has been shown to associate with left atrial dysfunction [19], [20]. Changes like increased left atrial volume index [27] may therefore also mediate the effect of SEC on thromboembolic events and mortality, which warrants further investigations including left atrial functional parameters.

Some strengths and limitations of our study deserve mentioning. The large sample size of 2400 patients provides statistical power to detect robust associations, enhancing the reliability of our findings. The comprehensive analysis, including multiple cardiovascular risk factors and their independent impacts on SEC, LAA thrombus, and mortality, offers a thorough understanding of the interplay between these variables. Additionally, the follow-up period with a median of 3.8 years allowed to assess longer-term outcomes. However, the observational design cannot reliably account for selection and indication bias and cannot establish causality. The findings from this single-center study may not be generalizable to other populations or healthcare settings. Furthermore, the lack of data on transthoracic echocardiography limits the association with functional parameters.

## 5 Conclusion

This study in patients with AF undergoing TEE highlights the relevance of SEC in the LAA as an independent predictor of both thromboembolic events and all-cause death, and underscores the importance of cardiovascular risk factors in predicting these findings. The results advocate cautious screening and management of AF patients with elevated risk profiles to mitigate adverse outcomes. Future prospective studies should validate these findings in diverse populations and explore the underlying mechanisms linking alkaline phosphatase to thrombus formation in AF patients.

## Patient and public involvement

Patients and/or the public were not involved in the design, or conduct, or reporting, or dissemination plans of this research.

## Patient consent for publication

Not applicable.

## CRediT authorship contribution statement

**Jan Traub:** Data curation, Formal analysis, Visualization, Writing – original draft. **David Hettesheimer:** Funding acquisition, Investigation, Writing – review & editing. **Jule Pinter:** Supervision, Writing – review & editing. **Floran Sahiti:** Formal analysis, Investigation, Writing – review & editing. **Georg Fette:** Conceptualization, Data curation, Formal analysis, Writing – review & editing. **Carsten Henneges:** Supervision, Validation, Writing – review & editing. **Caroline Morbach:** Data curation, Software, Supervision, Writing – review & editing. **Sebastian Herrmann:** Supervision, Validation, Writing – review & editing. **Frank Puppe:** Supervision, Validation, Writing – review & editing. **Anna Frey:** Supervision, Validation, Writing – review & editing. **Stefan Störk:** Project administration, Resources, Software, Writing – review & editing. **Martin Christa:** Conceptualization, Project administration, Resources, Supervision, Writing – review & editing.

## Ethics approval

The institutional review board of the University Würzburg approved the retrospective analysis of clinically acquired data (Corresp. 02022015). The study was conducted in accordance with the principles of the Helsinki Declaration.

## Funding

This work received public funding from the Bundesministerium für Bildung und Forschung (01ES0816, 01ES01901, 01ES01902, 01EO1004, and 01EO1504) and the German Research Foundation (391580509, 413657723, and 453989101).

## Declaration of competing interest

The authors declare that they have no known competing financial interests or personal relationships that could have appeared to influence the work reported in this paper.
